# Safety and Feasibility of Radiation Therapy Combined with CDK 4/6 Inhibitors in the Management of Advanced Breast Cancer

**DOI:** 10.3390/cancers15030690

**Published:** 2023-01-22

**Authors:** Marcin Kubeczko, Dorota Gabryś, Marzena Gawkowska, Anna Polakiewicz-Gilowska, Alexander J. Cortez, Aleksandra Krzywon, Grzegorz Woźniak, Tomasz Latusek, Aleksandra Leśniak, Katarzyna Świderska, Marta Mianowska-Malec, Barbara Łanoszka, Konstanty Chomik, Mateusz Gajek, Anna Michalik, Elżbieta Nowicka, Rafał Tarnawski, Tomasz Rutkowski, Michał Jarząb

**Affiliations:** 1 Breast Cancer Center, Maria Sklodowska-Curie National Research Institute of Oncology, 44-102 Gliwice, Poland; 2 Department of Radiotherapy, Maria Sklodowska-Curie National Research Institute of Oncology, 44-102 Gliwice, Poland; 3 Department of Biostatistics and Bioinformatics, Maria Sklodowska-Curie National Research Institute of Oncology, 44-102 Gliwice, Poland; 4 III Department of Radiotherapy and Chemotherapy, Maria Sklodowska-Curie National Research Institute of Oncology, 44-102 Gliwice, Poland

**Keywords:** abemaciclib, palbociclib, ribociclib, CDK 4/6 inhibitors, radiotherapy, advanced breast cancer

## Abstract

**Simple Summary:**

CDK4/6 inhibitors target cell proliferation resulting in toxicity, mainly bone marrow suppression manifesting as neutropenia. Radiation therapy (RT), widely used in breast cancer patients, may enhance myelotoxicity, and no consensus guidelines exist to guide practice when both treatments are to be instantiated. Since the risks of combination therapy are not well studied, many radiation and medical oncologists prefer to suspend CDK4/6i during RT. Nonetheless, a significant challenge that limits the efficacy of CDK4/6i treatment is the occurrence of acquired resistance that leads to the progression of metastatic disease. Thus, we performed a retrospective analysis of advanced breast cancer patients treated in a tertiary cancer center with RT sequentially or concomitantly to ribociclib, palbociclib, or abemaciclib. No excessive toxicities were observed after the addition of radiotherapy to systemic treatment. Our results may help to optimize multimodality treatment in a large population of patients with advanced breast cancer.

**Abstract:**

The addition of CDK4/6 inhibitors to endocrine therapy in advanced hormone receptor-positive HER2-negative breast cancer has led to practice-changing improvements in overall survival. However, data concerning the safety of CDK4/6i combination with radiotherapy (RT) are conflicting. A retrospective evaluation of 288 advanced breast cancer patients (pts) treated with CDK4/6i was performed, and 100 pts also received RT. Forty-six pts received 63 RT courses concurrently and fifty-four sequentially before CDK4/6i initiation (76 RT courses). Neutropenia was common (79%) and more frequent during and after concurrent RT than sequential RT (86% vs. 76%); however, CDK4/6i dose reduction rates were similar. In patients treated with CDK4/6i alone, the dose reduction rate was 42% (79 pts) versus 38% with combined therapy, and 5% discontinued treatment due to toxicity in the combined group. The risk of CDK4/6i dose reduction was correlated with neutropenia grade, RT performed within the first two CDK4/6i cycles, and more than one concurrent RT; a tendency was observed in concurrent bone irradiation. However, on multivariate regression analysis, only ECOG 1 performance status and severe neutropenia at the beginning of the second cycle were found to be associated with a higher risk of CDK4/6i dose reduction. This largest single-center experience published to date confirmed the acceptable safety profile of the CDK4/6i and RT combination without a significantly increased toxicity compared with CDK4/6i alone. However, one might delay RT for the first two CDK4/6i cycles, when myelotoxic AE are most common.

## 1. Introduction

Cyclin-dependent kinase 4 and 6 inhibitors (CDK4/6i) have been increasingly used in the setting of advanced hormone receptor (HR) positive, human epidermal growth factor receptor 2 (HER2) negative breast cancer, as well as in early disease [1]. In the advanced setting they constitute a mainstay of first/second-line therapies, as the addition of CDK4/6i to endocrine therapy (ET) provides a greater benefit in progression-free survival (PFS) and overall survival (OS) than endocrine therapy alone in patients with HR-positive, HER2-negative metastatic breast cancer (MBC) [2]. Furthermore, quality of life is either improved or maintained during this treatment [3].

The principal mechanism of CDK4/6i activity is the inhibition of Retinoblastoma (RB) protein phosphorylation [4]. CDK4/6i induce G1 cell cycle arrest and prevent the transition of the cell from G1 to S phase during the restriction point, with a consequent inhibition of cell cycle and proliferation. Since cells are more radioresistant during the S phase of the cell cycle, this mechanism of action may represent the cell radiosensitization potential of CDK4/6i. Pre-clinical data suggested a radiosensitizing effect and a potential synergy between CDK4/6i and radiation therapy (RT) [5,6]. Furthermore, in vitro analysis revealed that both the concurrent use of palbociclib with radiation, as well as palbociclib following irradiation, inhibited DNA double-strand break repair and promoted increased tumor cell apoptosis [7]. The complexity of the issue is further augmented by the fact that in the murine model, a protective effect of CDK4/6i on radiation-induced intestinal toxicity was shown [8].

To date, three CDK 4/6 inhibitors approved by the Food and Drug Administration (FDA) and the European Medicines Agency (EMA) are in widespread use, namely: palbociclib (since 2016), ribociclib (2017), and abemaciclib (2018). Despite similar clinical efficacy, these three compounds’ toxicity profiles vary with substantial pharmacokinetic differences [9]. Neutropenia is the most common adverse event observed in patients receiving palbociclib and ribociclib (all grades: 76.9%, median time to onset of G > 2 neutropenia: 16 days, average duration: 12 days) [10]. In contrast, abemaciclib less often induces neutropenia but at the cost of a higher frequency in G3–4 diarrhea (up to 19.7% compared with 4% for palbociclib) [11].

In contrast to previously standard endocrine monotherapy, CDK4/6i myelotoxicity profile raised safety concerns in the context of combining with RT. Grade 3–4 neutropenia (G3–4) occurred in 60%, and anemia G3–4 occurred in 3% of patients in a pooled analysis of MONALEESA trials [12]. Neutropenia was the main reason for dose reductions, and they were investigated in 818 patients who received ribociclib in combination with ET as first-line therapy across MONALEESA-2, -3, and -7 studies. Ribociclib dose reductions were required by almost half of patients (45.8%, range 37.0–57.5%), and 41.8% were a result of adverse events (AE), most commonly neutropenia. Severe adverse events led to treatment discontinuation in 14.6%, while progression-free survival was maintained in patients who received a decreased relative dose intensity due to AE. Furthermore, the overall response rate (ORR) and clinical benefit ratio (CBR) outcomes were not impaired in patients whose ribociclib dose was reduced due to AE compared with patients who received the full-dose regimen across the MONALEESA program. Similarly, in MONARCH 2 and MONARCH 3 trials, progression-free survival was also not impaired among patients with reduced doses [13].

Radiotherapy is one of the essential local control methods for symptomatic lesions in MBC. Up to 50% of patients with breast cancer will require palliative RT at some point during their disease course [14]. Furthermore, the role of ablative RT is becoming increasingly crucial in oligometastatic and oligoprogressive settings, taking into account the changing treatment landscape, improving the long-term prognosis of these patients and promising early results of such an approach. However, there is a paucity of data on the safety and feasibility of CDK4/6i treatment in combination with RT, and numerous questions regarding the toxicity of CDK4/6i combined with RT along with the optimal sequencing remaining unanswered. Although most published, mainly small, single-center studies showed encouraging results of the RT and CDK4/6i combination, some case reports warned about excess toxicity [15].

The most frequently seen toxicity with CDK4/6i treatment is myelotoxicity, and RT involving bone marrow may enhance myelosuppression. Additionally, delivering doses to the gastrointestinal (GI) tract may be an additional risk factor for overall toxicity [16]. This may lead to an avoidance of RT and preclude potentially beneficial treatment. Many radiation and medical oncologists prefer to suspend CDK4/6i during RT and base their approach on the extent and dosing of radiation therapy, sometimes also applying patient-related factors, e.g., the scale of myelosuppression induced by CDK4/6i. However, the acquired resistance leading to the progression of metastatic sites limits the overall efficacy of CDK4/6i, and radiotherapy shall augment this modality rather than lead to breaks in therapy. Thus, in the present study, we performed a retrospective data analysis of MBC patients treated in a tertiary cancer center with multimodality treatment in a real-world setting, either sequentially or concomitantly to ribociclib, palbociclib, or abemaciclib.

The primary endpoint of our study was CDK4/6i dose reduction in patients treated with CDK4/6i and RT. The secondary endpoints were hematological and non-hematological toxicity grade ≥ 3 and CDK4/6i discontinuation rates. All these endpoints were compared between patients receiving CDK4/6i concurrently with RT and patients receiving CDK4/6i sequentially after RT completion.

## 2. Materials and Methods

### 2.1. Study Group—Patient Selection

**Figure 1:**
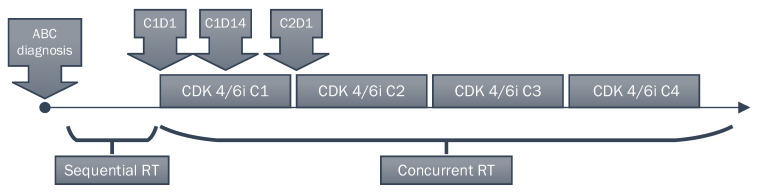
Study schema. Abbreviations: ABC—Advanced breast cancer; CDK4/6i—Cyclin-dependent kinase 4 and 6 inhibitors; C—Cycle; C1D1—First day of the first cycle of CDK4/6i treatment; RT—Radiation therapy.

Records of consecutive patients with advanced breast cancer who received palbociclib, ribociclib, or abemaciclib in our institution were reviewed since the initiation of CDK4/6i therapy in Poland (2017) until the beginning of 2022. Both the timing of CDK4/6i therapy and all courses of radiation therapy were recorded. Subsequently, patients who received CDK4/6i treatment with sequential or concurrent external beam radiation therapy were enrolled in this study. The majority of patients underwent systemic treatment in the Breast Unit, while radiation therapy procedures were carried out in the Radiotherapy Department. Thus, all data collected were derived from a real-life setting, and no supplementary visits associated with the study were performed. Concurrent use was defined as the initiation of radiation therapy after the first day (cycle 1 day 1, C1D1) of CDK4/6i treatment. For sequential therapy, only patients treated by radiation before initiation of CDK4/6i were included to avoid multiple factors complicating the patient situation after progression (next lines of systemic therapy or performance status deterioration). The temporal relationship between RT and CDK4/6i treatment, as defined in the study, is shown in Figure 1.

National Cancer Institute Common Terminology Criteria for Adverse Events (NCI-CTCAE) v5.0 was used to assess toxicity. The primary endpoint of our analysis was the impact of RT on CDK4/6i dose reduction, and secondary endpoints include CDK4/6i discontinuation, adverse events grade ≥ 3, and neutropenia (any grade).

All procedures performed in the study were in accordance with the 1964 Helsinki declaration with later amendments and with the ethical standards of the institutional Ethics Committee, which approved the study (no. KB/430-27/22).

### 2.2. Treatment

#### 2.2.1. CDK4/6 Inhibitors Treatment

All the drugs were administered according to product specifications. Ribociclib was given at the dose of 600 mg once daily; 3 × 200 mg tablets q.d.; 21 consecutive days of treatment, followed by seven days break. Palbociclib was used initially at the dose of 125 mg once daily; 1 tablet q.d.; 21 consecutive days of treatment, followed by 7 days break. Abemaciclib was given at 150 mg twice daily; 1 tablet b.i.d.; 28 consecutive days of treatment continuously. Dosage reductions were allowed at the prescriber’s discretion based on toxicities (ribociclib first reduction level to 400 mg, second reduction level to 200 mg daily; palbociclib first reduction level to 100 mg daily, second reduction level to 75 mg daily; abemaciclib first reduction level to 200 mg daily and second reduction level to 100 mg daily). All three CDK4/6i were given in association with either letrozole (2.5 mg tablet; once daily throughout the 28-day cycle) as first-line treatment for MBC or with fulvestrant (500 mg every 28 days) as first or second-line treatment for MBC. Premenopausal patients received additional Luteinizing Hormone-Releasing Hormone-agonists (LHRH-agonists) for ovarian function suppression.

#### 2.2.2. Radiation Therapy

In the majority of patients, radiotherapy was delivered with palliative intent due to symptomatic bone metastases. Three-dimensional conformal radiotherapy (3D-CRT), volumetric modulated arc therapy (VMAT), and intensity modulated radiation therapy (IMRT) with moderate hypofractionation were the most common techniques used to treat bone metastases. Stereotactic or radiosurgery treatment was applied in some patients with oligometastatic disease. Those patients received more precise treatment with higher doses. RT was performed with Varian Truebeam^®^ Linear Accelerator (Varian Medical Systems, Palo Alto, CA, USA), Cyberknife^®^ (Accuray Incorporated, Sunnyvale, CA, USA), or TomoTherapy^®^ System (Accuray Incorporated).

Gross tumor volume (GTV) was defined individually, generally including the macroscopic tumor volume or osteolytic lesion. Additional margins were added for the possible extension to the adjacent tissues to contour clinical target volume (CTV). In the postoperative setting, e.g., in brain metastases, no GTV was contoured, and CTV was delineated as a tumor bed. Planning target volume (PTV) was outlined, adding an adequate margin to the CTV. In stereotactic radiotherapy CTV was equal with PTV. The prescribed dose was defined according to guidelines for palliative radiotherapy and personalized with respect to patients’ characteristics, such as tumor site and burden, previous treatments, and performance status. Throughout RT, every patient was monitored by a radiation oncologist at least once a week for evaluation and management of early toxicity. 

### 2.3. Statistical Analysis

Categorical variables were summarized as frequencies and percentages. Pairwise comparisons between patient subgroups were performed by Fisher’s exact test or Fisher–Freeman–Halton test, and odds ratios (OR) were calculated.

Continuous data were shown as median values with interquartile ranges (25 to 75%, IQR 25–75) and mean values with standard deviation/min/max ranges. Data were analyzed using parametric and nonparametric methods depending on distribution and homogeneity of variance. Differences between the two groups were determined using the Wilcoxon rank sum test and the two-sample *t*-test. The Cochran–Armitage test was applied for trend analysis, and Spearman’s rank correlation coefficient was assessed as an effect size parameter. Hazard ratios (HRs) with 95% confidence intervals (CIs) were estimated by univariate and multivariate Cox’s proportional hazards regression models. Variables with *p*-value  <  0.20 in the univariate analysis were included in the multivariate analysis.

All analyses were performed using the R environment for statistical computing version 4.0.2 “Taking off Again” released on 22 June 2020 (R Foundation for Statistical Computing, Vienna, Austria, http://www.r-project.org (accessed on 29 February 2000). A two-sided *p*-value < 0.05 was considered statistically significant, and a *p*-value < 0.10 was considered close statistical significance.

## 3. Results

### 3.1. Study Population

Two hundred eighty-eight patients with ER positive HER2 negative advanced breast cancer were treated with CDK4/6 inhibitors in our center from November 2017 to February 2022 (149 pts with ribociclib, 117 with palbociclib, and 22 with abemaciclib; 285 women and three men). Forty-two patients started treatment with CDK4/6i from November 2017 to the end of 2019. After the reimbursement of these drugs in Poland, 120 patients began CDK4/6i treatment in 2020, 124 patients in 2021, and two pts in January 2022. Among these patients, 100 received CDK4/6i treatment and RT and were further analyzed.

The median age of the patients was 58.5 years (range min/max: 23–83 years), including 29 pts under 50 (29%), 37 pts were diagnosed with de novo metastatic disease, whereas 63 patients had disease recurrence. In total, 56% of patients had received previous chemotherapy, including 10% with chemotherapy courses finished within one year before the beginning of CDK4/6i treatment. In total, 52% of patients had a bone-only disease, 45% had metastases in bones and other localizations (soft tissues, lymph nodes, visceral), 3% had a visceral-only disease, and 11% of patients had brain metastases. Most patients were treated in the first-line setting of an advanced disease (69%) and received letrozole as a hormonotherapy backbone (65%), while fulvestrant was used in 4% of patients treated in the first line and all patients in the second-line setting (31%). In total, 65% of patients received ribociclib, 27% palbociclib, and 8% abemaciclib. Baseline patients’ characteristics with a comparison between patients with concurrent and sequential RT are shown in Table 1. Both groups were well-balanced, with no significant differences among them.

### 3.2. Radiation Therapy

As the majority of patients were treated with palliative intent, the most often used schema was 20 Gy delivered in five fractions. In stereotactic radiotherapy group treatment was usually delivered with three fractions every other day. Radiotherapy treatment details, including doses, are summarized in Table 2.

Bone metastases were predominantly localized with the highest number in the vertebrae (Table 3). In three patients with multiple bone metastases LHBI or MHBI was performed.

One hundred thirty-nine radiation treatments were performed in 100 patients. Forty-six patients received RT concurrently with CDK 4/6 inhibitors treatment. Fifty-four patients received sequential radiotherapy only. Seventy-six sequential RT were performed in the majority of patients as single course, but there were patients with three radiotherapy courses. In some of the patients (12 pts) both sequential and concurrent RT courses were performed. Those patients were analyzed within the concurrent RT arm. Patients who received RT more than six months before the start of CDK4/6i treatment were not included in the analysis.

Half of the sequential treatments were performed within four weeks before the start of CDK4/6i treatment. The initiation of CDK4/6i treatment started as early as 1 week after RT courses and up to 12 weeks. The courses distribution was as follows: within 1 week (10 RT courses); >1–2 weeks (7 RT courses); >2–4 weeks (21 RT courses); >4–8 weeks (16 RT courses); >8–12 weeks (13 RT courses); and 12 weeks <six months (9 RT courses).

Sixty-three concurrent RT treatment courses were performed in 46 patients. In sequential treatment, courses of radiotherapy were performed mostly in one but in up to 5 concurrent courses. Almost half received RT during the first cycle of CDK4/6i treatment (20 pts, 44%; 27 treatments), while others received RT during later cycles. Patients receiving RT concurrently were receiving a full dose of CDK4/6i in most cases (35 pts). Eight patients were irradiated with the first level of CDK4/6i reduction and three with the second level of reduction. 

### 3.3. Safety

Hematological toxicity was the most common reason for the observed adverse events of the therapy. Neutropenia was most prevalent, and the majority of patients experienced it at some point during the study period (79%), with rates comparable in concurrent and sequential arms (83% and 76%, respectively, *p* = 0.3). Neutropenia occurred in one-quarter of pts during RT (23%) and a half after RT (54%). However, when analyzed in a close temporal relationship to radiation, neutropenia was found to be more frequent during and after concurrent RT than sequential RT (*p* < 0.001).

**Figure 2:**
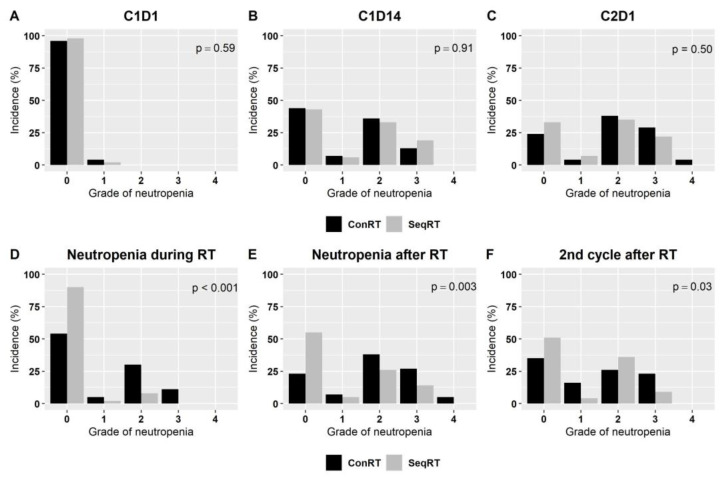
Rates of neutropenia in C1D1 (**A**), C1D14 (**B**), C2D1 (**C**), during RT (**D**), shortly after RT (**E**), and in the second cycle after RT (**F**) in the group with sequential radiation therapy (SeqRT) and concurrent radiation therapy (ConRT). 0—no neutropenia; 1—neutropenia G1; 2—neutropenia G2; 3—neutropenia G3; 4—neutropenia G4.

The rates of neutropenia in C1D1, C1D14, and C2D1 of CDK4/6i treatment were similar in both groups (n.s., *p* = 0.59, *p* = 0.91, and *p* = 0.49, respectively). However, after RT, neutropenia was more pronounced in the second CDK4/6i cycle (*p* = 0.03). Rates of neutropenia in C1D1, C1D14, and C2D1, during RT, shortly after RT, and in the second cycle after RT, are shown in Figure 2.

Rates of thrombocytopenia on C1D1 were similar between groups (*p* = 0.74). However, anemia on C1D1 was more pronounced in the concurrent RT arm (*p* = 0.04). Within the whole group of patients, two patients developed G3 thrombocytopenia and one G4 thrombocytopenia after RT treatment. One patient had G3 anemia after RT, and no cases of G4 anemia were reported.

Almost all radiation therapy courses in concurrent treatment were performed as planned, without suspensions or dose reductions. Early radiation toxicity was limited and represented mainly by grade 1–2 reactions, except for fatigue and skin toxicity. One patient had G3 fatigue lasting 10 days after stereotactic RT for a tumor bed in the cerebellum with 24 Gy delivered in three fractions. Only one patient had to stop the RT course earlier (after 27 of 30 Gy). She was irradiated for metastases in the thoracic spine; the treatment was interrupted because of worsening fatigue (from G2 to G3) and bone pain. One patient had G3 skin toxicity in the radiation field after 50 Gy delivered in 25 fractions for breast and regional lymph nodes irradiation; this was not considered an augmented reaction. Moreover, ribociclib treatment started within one week of RT completion and did not worsen her skin toxicity resolution. No grade > 2 early or late radio-induced gastrointestinal, neurological, or other radiation-induced toxicities were observed. Diarrhea G1–2 occurred in six out of eight patients treated with abemaciclib, with no G3–4 episodes.

With a median follow-up of 17 months after completion of RT, no patient experienced any severe late toxicity. No dose-response effect was observed for the potential toxicity of radiation. The majority of patients irradiated for bone metastases experienced pain relief.

#### 3.3.1. CDK4/6i Dose Reductions

Overall, 38 patients had CDK4/6i dose reduction (38%): 16 patients (35%) in sequential-only RT and 22 patients (41%) in the concurrent RT group (difference not significant, n.s., *p* = 0.07). However, eight patients in the concurrent arm were already on a reduced dose of CDK4/6i prior to RT, due to previous medication-related toxicity (with that adjustment, *p* = 1.0).

More than half of the patients who required CDK4/6i dose reduction had dose reduction during the second or third CDK4/6i cycle (22 patients, 22% overall). In total, 30 pts had CDK4/6i dose reduction after RT (range 1–12 cycles after RT), including 19 pts who had CDK4/6i dose reduction in the first cycle following RT (seven patients), in the second cycle after RT (eight patients), in the third cycle after RT (one patient), and in the fourth cycle after RT (three patients). Among these 19 patients, 9 (20%) were treated with concurrent RT and 10 (19%) with sequential RT (*p* = 1.0). The remaining patients had CDK4/6i dose reduction in later cycles after RT (5 cycles or more after RT, 11 pts). The median time to CDK4/6i reduction was similar in both groups (*p* = 0.81).

The vast majority of patients (33 out of 38) had CDK4/6i dose reduction due to neutropenia. The other five patients had CDK4/6i dose reduced because of hepatotoxicity, pulmonary toxicity, and vomiting. Three patients who had their doses reduced due to hepatotoxicity all received ribociclib. RT was performed in these patients outside the liver: the thoracic wall, breast and regional lymph nodes, and lumbar spine, respectively. One patient had the dose reduction during the sixth cycle of CDK4/6i because of post-COVID-19 inflammatory interstitial lung disease. He received thoracic spine irradiation with 8 Gy delivered in a single dose eight months earlier. One patient had their dose reduced as a result of prolonged G2 nausea/vomiting before the start of RT. The causes of CDK4/6i dose reductions were equally distributed between the groups (*p* = 0.53).

CDK4/6i dose reduction was not correlated with radiation dose in the sequential and concurrent RT groups (*p* = 0.12). The time from irradiation to the start of CDK4/6i treatment was also irrelevant, even when sequential RT was completed just before CDK4/6i initiation (*p* = 0.35). There was a trend towards more frequent dose reductions in patients receiving more than one sequential RT treatment (*p* = 0.059), and more than one concurrent RT correlated with a higher risk of CDK4/6i dose reduction (*p* = 0.015). The site of irradiation was not associated with CDK4/6i dose reduction in sequential treatment; however, in the concurrent group a tendency toward more frequent reductions in patients receiving bone irradiation was observed (*p* = 0.051). The CDK4/6i dose reduction risk was also higher when radiation was performed within the first two CDK4/6i treatment cycles (*p* = 0.027; odds ratio OR 9.0). 

Neutropenia was the strongest factor associated with CDK4/6i dose reduction. Most patients had at least one episode of neutropenia (79%) during the study, which was associated with CDK4/6i dose reduction (*p* = 0.04). Patients with any grade neutropenia in the beginning of CDK4/6i treatment: C1D1, C1D14, or C2D1 had a higher risk of CDK4/6i dose reduction (*p* = 0.006; *p* < 0.0001; *p* < 0.0001, respectively). The neutropenia grade correlated with higher risk, and patients with severe neutropenia (G3–4) in C1D14 or C2D1 had a higher risk of CDK4/6i dose reduction (*p* < 0.0001; OR 9.1; *p* < 0.0001; OR 13.9, respectively). 

Four RT treatments were performed during G1 neutropenia, 22 during G2 neutropenia, and seven during G3 neutropenia. Radiotherapy during severe neutropenia (G3) was associated with CDK4/6i dose reduction (*p* = 0.001; OR 20.6). Neutropenia shortly after RT and in the 2nd cycle after RT was associated with CDK4/6i dose reduction (*p* = 0.0007; OR 5.5; *p* = 0.0002, respectively), especially G3-G4 neutropenia (*p* < 0.0001; OR 25.2; *p* < 0.0001; OR 7.8). Thrombocytopenia after RT completion was also correlated with CDK4/6i dose reduction, especially G > 1 (*p* = 0.004; *p* = 0.006; OR 10.4, respectively). 

On Univariate Cox’s proportional hazards regression model, CDK4/6i dose reduction was not associated with age, de novo or recurrent disease, previous chemotherapy, CDK4/6i type, concurrent vs. sequential RT, line of treatment, and bone RT. CDK4/6i dose reduction was found to be associated with ECOG performance status (ECOG 0 vs. ECOG 1), neutropenia in C1D14 (G0 vs. G3–4), and neutropenia in C2D1 (G0 vs. G3–4). Variables with *p* ≤ 0.2 were selected for multivariable analysis (age, ECOG performance status, concurrent vs. sequential RT, RT area, neutropenia in C1D14, and neutropenia in C2D1). Multivariate analysis showed that ECOG 1 performance status and neutropenia in C2D1 (G3–4) increased the risk of CDK4/6i dose reduction with HR = 3.13 and HR = 16.11, respectively. Results are shown in Tables S1 and S2. Additionally, among all 288 breast cancer patients treated at our center with or without additional irradiation, 117 required CDK4/6i dose reduction (41%). In patients treated with CDK4/6i only (without RT), the dose reduction rate was 42% (79 patients) versus 38% in patients with combined therapy (*p* = 0.53).

#### 3.3.2. CDK4/6i Treatment Discontinuation

At the time of data cut-off, 40 patients (40%) discontinued CDK4/6i treatment, mainly due to disease progression (75% of discontinuations, 30 patients). The reasons for discontinuation were similar in both arms (*p* = 0.86). Five patients (5%) discontinued treatment due to the toxicity of CDK4/6i treatment: two due to hepatotoxicity, two as a result of hematologic toxicity (one neutropenia, one thrombocytopenia), and in one patient the reason was not apparent (follow-up lost). In patients with hepatotoxicity, radiation was performed on the area outside the liver (thoracic and lumbar spine). One patient discontinued ribociclib one year after bone RT to the thoracic vertebrae due to unclear inflammatory disease with the swelling of several joints, a rash, and pruritus (no complete follow-up of the diagnostic process at the time of manuscript preparation). Discontinuations due to myelotoxicity were rare (two patients). One patient discontinued CDK4/6i treatment after three cycles due to prolonged G3 neutropenia despite dose reductions. She was treated for metastases in the thoracic vertebrae with 20 Gy in five fractions and commenced irradiation three days before ribociclib C1D1; then she received irradiation with 8 Gy in one fraction for the femur and 20 Gy in five fractions for lumbar and sacral bone during the first cycle. Grade 3 neutropenia persisted for 3 weeks. The second patient developed thrombocytopenia G4. She received lower hemi-body irradiation 8 Gy in one fraction two months before discontinuation. However, heparin-induced thrombocytopenia cannot be excluded as the reason for severe thrombopenia, as the patient concomitantly received low-molecular-weight heparin in therapeutic doses for the treatment of pulmonary embolism; this treatment was temporarily modified, concurrently with platelets transfusion. 

The reason for CDK4/6i discontinuation was unknown in two patients—patients were lost to follow-up. One patient died after spine surgery. Two patients discontinued treatment due to a gradual worsening of performance status, both with CNS involvement. One lady received whole brain radiotherapy with 20 Gy in five fractions during the seventh cycle of ribociclib treatment and her performance status worsened six months later. She had epidural involvement at the time of breast cancer diagnosis and severe chronic obstructive pulmonary disease. Because of severe cachexia and weight loss (BMI 11.3), she discontinued ribociclib and continued letrozole monotherapy. In the second patient, radiation was performed sequentially and commenced five months before the first cycle of abemaciclib. After two cycles of abemaciclib, treatment was discontinued due to worsening overall performance status.

### 3.4. Treatment Outcomes

The majority of patients (86%) achieved a clinical benefit (23% partial response, 63% stable disease), and 9% had disease progression as the best overall response. Results were similar in both groups (*p* = 0.62). In five patients, data concerning response were missing, or response assessment was not performed due to early patient status deterioration. At a median follow-up of 17 months, local control was achieved in 90% of patients (five patients had disease progression in a previously irradiated site, and in five patients’ data were missing). There was no difference between the concurrent and sequential RT groups (*p* = 0.65).

## 4. Discussion

The combination of CDK4/6 inhibitors with endocrine therapy in advanced HR+/HER2− breast cancer is the mainstay of the initial therapy of this disease. Radiotherapy’s role is crucial in the locoregional management of metastatic disease, and no consensus guidelines exist to support the combination of both modalities. Our analysis, with 100 patients and 139 RT treatments, is the largest single-center experience published to date and it confirmed no clinically significant increase in early or late toxicities with CDK4/6i and radiotherapy combination.

CDK4/6i target the cell cycle with a bone marrow suppression effect as the primary dose-limiting toxicity; the occurrence of neutropenia is rather a rule—in our study 79% of patients experienced at least one episode, consistent with meta-analysis that reported all-grade neutropenia frequency of up to 80% [17]. Other myelotoxicity episodes were extremely rare—severe thrombocytopenia was reported in three patients (3%) and anemia in one patient (1%). Other publications report a G3–4 thrombocytopenia rate ranging from 0.6 to 3.4% and G3–4 anemia from 1.2 to 7.2%, placing the thrombopenia rate in our study in the upper range and anemia occurrence in the range of lower frequency.

Likewise, in our study, 38% of patients required CDK4/6i dose reduction. The CDK4/6i dose reduction risk was higher when radiation was performed within the first two CDK4/6i treatment cycles (*p* = 0.027; OR 9.0) and when more than one concurrent RT treatment was performed (*p* = 0.015).

In MONALEESA trials, the ribociclib dose was reduced in 45.8% of patients [12]. Likewise, in our study, 38% of patients required CDK4/6i dose reduction. Discontinuation rates due to AEs were reported in MONALEESA trials in 14.6% of patients [12]. Other meta-analyses reported CDK4/6i dose reduction rates ranging from 31.6 to 53.9%, and drug discontinuation due to toxicity ranging from 2.6 to 19.6%. Similarly, in our study, patients who discontinued CDK4/6i treatment for a reason other than a progression of the disease comprise 10% of patients.

Norman et al. [18] presented a retrospective single-institution experience characterizing 47 patients who received palliative radiation to bony metastases within one year before starting palbociclib. There were comparable rates of neutropenia, anemia, and thrombocytopenia. In line with this study, we observed that hematologic toxicity after RT was not associated with CDK4/6i treatment modification.

Several studies have shown that reducing the dose of CDK4/6i due to toxicity, such as neutropenia, did not worsen the efficacy of the treatment, and patients with a reduced dose of CDK4/6i responded as well as patients with a full dose [12,13]. However, these results concern patients with dose reductions due to CDK4/6i toxicity, which may not comprise all causes of dose reductions, such as toxicity induced by other treatment modalities. As a result, the addition of other treatment modalities, such as radiotherapy, should be analyzed if they do not increase dose reductions and discontinuations.

The early adverse events of the concomitant use of CDK4/6i and radiation therapy in sixteen patients with metastatic breast cancer were summarized by Ippolito et al. [19] in 2019. The most common toxicity was neutropenia, including G3 neutropenia in 25% of patients. Only one patient (6.3%) developed G4 neutropenia and required CDK4/6i dose reduction. In our study, CDK4/6i dose reductions rates were higher due to a longer follow-up, since, e.g., recurrent G3 neutropenia also requires dose reduction.

Furthermore, in 2019 a case report about enhanced dermatologic reaction after the combination of palbociclib and curative-intent radiation therapy was published [20]. Severe skin toxicity was also reported by Harvey-Jones et al. [21] in a retrospective on 23 patients treated with CDK4/6i and 28 courses of RT. Furthermore, Beddok et al. [22] found one episode of G3 dermatitis after concurrent treatment with palbociclib and locoregional irradiation. 

Similarly, we observed only one severe skin toxicity. The patient developed G3 dermatitis in the radiation field after 50 Gy delivered in 25 fractions for breast and regional lymph nodes irradiation. Treatment with ribociclib was started within one week after RT completion and did not worsen skin toxicity resolution. 

Grade 3 skin toxicity was also reported in two patients by Howlett et al. [23]. They published data from 42 radiotherapy courses while established on, or within 28 days of commencing, CDK4/6i. Though, for the majority (88%) of RT series affected by toxicity, the maximum grade was 1–2 and did not lead to CDK4/6i treatment alterations. Hematological side effects did not seem worsened by radiotherapy delivery. Likewise, we observed comparable rates of hematological toxicity in patients with sequential RT, concurrent RT, or without any RT.

Preliminary data with ribociclib published by Meattini et al. in 2018 showed encouraging results [24]. However, G ≥ 2 diarrhea and vomiting were reported in a patient after hip radiation. Some data suggested over-sensitization secondary to CDK4/6i administration resulting in severe gastrointestinal toxicity after RT. Guerini et al. [25] published outcomes of 18 MBC patients treated to 32 sites concomitantly with CDK4/6i. Early toxicity was generally limited and represented mainly by G1 toxicity, except for G3 radiation-induced ileitis during palbociclib treatment. Severe acute radiation-induced enterocolitis after concurrent palbociclib and radiation therapy treatment was also reported by Kawamoto et al. [16]. It is worth noting that, in our study, the irradiated sites included 22 sites in the pelvis, half concurrently with CDK4/6i and half sequentially. Conversely, no severe early or late gastrointestinal toxicity after RT was observed. One patient in our cohort required CDK4/6i dose reduction due to vomiting; however, it occurred before the start of RT. Up to 86.4% of patients treated with abemaciclib experienced diarrhea, the most common adverse event associated with this CDK4/6i [26]. In our study, diarrhea G1–G2 occurred in 75% of patients treated with abemaciclib (6 out of 8), but no episodes of severe diarrhea were reported.

CDK4/6i are associated with a significant risk of severe hepatic dysfunction which was reported in 4.1% of patients [27]. In our study, hepatotoxicity led to CDK4/6i dose reduction in 3% of patients and treatment discontinuation in 2% of patients. Livers’ function tests must be systematically monitored for prompt detection and proper management. CDK4/6i significantly increased the risk of interstitial lung disease/pneumonitis, with an overall incidence of 1.6% [28]. In our study, one patient (1%) had a dose reduction due to inflammatory interstitial lung disease; however, it occurred after a COVID-19 infection. This patient had received thoracic spine irradiation with 8 Gy delivered in a single dose eight months earlier.

Hans et al. published the preliminary results of the correlation between radiotherapy and palbociclib [29], including radiosurgery for liver metastases in one case (60 Gy/10 fractions). We performed stereotactic body radiation therapy for liver metastases with 45 Gy delivered in three fractions concurrently with CDK4/6i in two patients. Treatment was also well tolerated, with no new safety signals.

Among a cohort of 46 patients who underwent 62 metastases-directed RT, reported by Ratosa et al. [30], the decreased white blood cell and neutrophil count were the most frequent severe AE; all except one severe AE were hematological. Type of CDK4/6i, CDK4/6i suspension during RT, planning target volume, total prescribed dose of RT, nor RT technique did not worsen the cumulative toxicity of any grade.

A recent review of literature by Bosacki et al. [31] on CDK4/6i combined with radiotherapy in an advanced setting was published. They analyzed seven publications describing the outcome of 92 radiotherapy procedures combined with CDK4/6i in 59 patients. They suggest CDK4/6i suspension for five half-lives before and after radiotherapy. However, suspension of treatment would range from approximately 12 days in RT involving only one fraction to more than six weeks in the case of irradiation of the breast or chest wall with a dose of 50 Gy delivered in 25 fractions. There is little evidence that such a suspension would not diminish the efficacy of CDK4/6i treatment.

However, the issue is not fully clear and in the setting of early breast cancer, abemaciclib was administered as an adjuvant therapy after the completion of postoperative radiation therapy, and no concurrent treatment was allowed (monarchE study, [1]). 

The majority of the serious adverse events reports are from case reports from which, however, we can learn about specific treatment toxicity [32]. For example, in the case of radiotherapy within the mediastinum and right hilum with 17 × 3 Gy, prolonged G2 dysphagia with a G2 esophageal ulcer was reported, despite palbociclib being stopped 3–4 days before RT and being restarted 8–9 days after RT [15]. It is difficult to state that this was related to the combined treatment or that radiotherapy itself caused such a toxicity.

Concerningly, more case reports have been published describing various toxicities [15], though results from larger cohorts were more reassuring. Kim et al. published a recent retrospective analysis of thirty BC women who received 36 palliative RT courses within 14 days of CDK4/6i use [33]. No patients required dose reduction of CDK4/6i after RT, though four patients were already on a reduced dose of palbociclib prior to RT due to previous toxicity. No G ≥ 3 constitutional, gastrointestinal, or neurologic toxicities were reported. However, in this study, only eight patients received CDK4/6i concurrently, and in a majority of patients, CDK4/6i treatment was started after RT or was stopped prior to RT. Five patients underwent brain radiotherapy. A group of patients in our study also had CDK4/6i dose reduction prior to RT due to previous drug toxicity (8% of patients). Likewise, in our cohort, no serious gastrointestinal toxicity was observed. Moreover, 11 patients underwent brain radiotherapy without severe neurologic toxicity. This is consistent with other studies concerning brain metastases management. Figura et al. [34] published a retrospective evaluation of the clinical outcomes of breast cancer patients treated with CDK4/6i and stereotactic radiation in the management of brain metastases. Radionecrosis developed in two lesions (5%) in patients with four prior RT courses. Results from our study confirm that CNS radiation in the context of CDK4/6i treatment is a safe option—eleven patients were irradiated without significant toxicity.

Chowdhary et al. [35] reported outcomes of 16 breast cancer patients treated with RT for symptomatic metastasis concurrently or within 14 days of palbociclib administration. No new or late toxicity was reported at the median follow-up of 14.7 months. There is a paucity of data concerning the late toxicity of CDK4/6i and RT combination. Correspondingly, in our study with a comparable follow-up, comprising all three approved CDK4/6i, we did not find any clinically significant late or new toxicity, and no difference regarding the irradiated site was observed. However, there was a trend toward a higher risk of CDK4/6i dose reduction in patients treated for bone metastases.

Meattini et al. [36] recently analyzed a large cohort of 85 consecutive patients treated with CDK4/6i, among which 25 (29.4%) received metastases-directed RT, including 14 concomitant (16.5%) treatments. At a median follow-up time of 12 months, 35 patients (41.2%) required dose reduction, and five patients (5.9%) discontinued treatment due to AE. There was no significant difference in CDK4/6i dose reduction, discontinuation, or high-grade toxicity in the comparison between patients receiving RT versus no RT and between patients receiving concomitant RT versus sequential RT versus no RT.

The largest cohort of patients was published recently by Al-Rashdan et al. [37]. One hundred thirty-two patients treated with CDK4/6i in combination with RT (220 RT sites) were compared with 53 patients treated with RT alone (93 sites). Early toxicity was predominantly found in the combined treatment group. However, it was not significant in multivariable analysis after propensity score matching. No significant association between CDK4/6i use and early ≥G2 nonhematological toxicity was found, which aligns with our results.

Patients with HR+/HER2− advanced breast cancer have a relatively long overall survival and many treatment possibilities; thus, the effect of both therapy and the disease on the patient’s symptoms and functioning is crucial [38] Health-related quality of life (HR-QoL), usually assessed with the patient-reported outcome measures (PROM) has gained an increasing value when evaluating cancer therapies. Data from the literature were reassuring—CDK4/6i did not worsen patients’ HR-QoL compared with endocrine therapy alone [39]. Pain is an important symptom covered by EORTC QLQ-C30 [40]. Addressing bone pain management influencing lower QoL might improve the QoL of breast cancer patients. RT effectively relieves pain from bone metastases as a single fraction of 8 Gy [41]. However, fractionated regimens, such as 20 Gy in five fractions might be an option for pts with a relatively long-life expectancy since there is a decreased need for retreatment [42]. Given data on the overall survival benefits with CDK4/6i treatment [43], the addition of another treatment modality should not interfere with this therapy. In our study, the majority of patients experienced bone pain relief after palliative RT with no enhanced toxicity.

Currently, we await the results of prospective data from phase II studies concerning a combination of CDK4/6i treatment and RT in specific populations: CLEAR trial (NCT03750396), ASPIRE trial (NCT03691493), and PALATINE trial (NCT03870919). AVATAR is another ongoing multi-centre phase II trial of stereotactic radiotherapy with CDK4/6i treatment in advanced breast cancer and aims to enroll 32 patients with oligoprogressive disease limited to five lesions [44]. In our study, 32 patients received stereotactic radiotherapy, and this combination emerged safe and well tolerated.

To our knowledge, our study is the largest single-center experience published to date with the second-largest cohort. The study was conducted in one cancer center.

Furthermore, the strength of this study is that patients were assessed for toxicity not at a single time point after RT but on a regular basis. As a result, the rates of toxicity, driven mainly by CDK4/6i myelotoxicity, resembles the rates of adverse events in the main clinical trials with CDK4/6i. Moreover, our study provided an extended follow-up comprising all three approved CDK4/6i. No new toxicities were observed in longer follow-up except in those characteristic of CDK4/6i.

Our results confirm, on a larger cohort, the feasibility and safety of the concurrent treatment with no additional toxicity. RT did not appear to have an additive effect on myelosuppression. Nevertheless, patients during RT should be meticulously monitored, especially in the first cycles of CDK4/6i treatment, since severe hematologic toxicity is most likely to occur at the beginning of the treatment.

We acknowledge the inherent limitations of a retrospective study design. However, despite the retrospective character of the study, groups of patients with sequential and concurrent RT appeared to be well-balanced, supposedly due to relatively large cohorts.

There are some pitfalls of our study. Except for neutropenia, we focused on severe adverse events (toxicity grade 3–4), toxicities that led to the suspension of radiotherapy or CDK4/6i, and toxicities leading to dose reduction or treatment discontinuation (either RT or CDK4/6i). We did not include in the analysis, e.g., mild gastrointestinal toxicity. Furthermore, patient-reported outcome measures would be important in this scenario.

Twelve patients underwent both sequential and concurrent RT. They were analyzed in the concurrent RT group since it seems to be associated with more toxicity than sequential RT. Nevertheless, toxicity was not higher in the concurrent group, even when preceded by sequential RT.

Heterogeneity in RT regimens, fractionation, doses, technique, and treatment at various time-points of CDK4/6i treatment are also limitations of our study, however, this reflects daily clinical practice.

## 5. Conclusions

This largest single-center experience published to date confirmed the acceptable safety profile of the combination CDK4/6i and RT, with no unexpected toxicities. CDK4/6i and RT combination seems well tolerated and could be used safely since no significant increase in early or late toxicities was reported compared with when CDK4/6i were administered alone. CDK4/6i dose reduction was similar in patients with concurrent and sequential RT, and rates were comparable with patients who did not receive RT. These results are encouraging since neutropenia is common with CDK4/6i, and RT can also diminish neutrophils’ levels. High-grade hematological toxicity was frequent, but the addition of radiotherapy did not change the treatment course in the majority of patients. Given this experience, CDK4/6i do not have to be suspended during palliative RT. However, these results have to be confirmed in a larger cohort.

## Figures and Tables

**Table 1:** Baseline patients’ characteristics divided into a group with concurrent radiation therapy vs. sequential only radiation therapy.

Characteristics	Concurrent RT (*n* = 46)	Sequential RT (*n* = 54)	*p*-Value
Age (median (IQR))	58 (49–66.75)	59 (48–65.75)	0.39
Age < 50 yo (*n* (%))	13 (28.26%)	16 (29.63%)	1.00
De novo disease (*n* (%))	19 (41.3%)	18 (33.3%)	0.53
ECOG 0 (*n* (%))	17 (37%)	19 (35%)	
ECOG 1 (*n* (%))	22 (48%)	24 (45%)	0.83
ECOG 2 (*n* (%))	7 (15%)	11 (20%)	
1st line treatment (*n* (%))	32 (69.57%)	37 (68.52%)	1.00
Localization of mets Bone only (*n* (%)) Bone + other (*n* (%)) Visceral only (*n* (%))	24 (52%) 20 (44%) 2 (4%)	28 (52%) 25 (46%) 1 (2%)	0.81
Brain metastases (*n* (%))	2 (4.4%)	9 (16.7%)	0.14
Previous CHT (*n* (%))	23 (50%)	33 (61.11%)	0.32
CHT < 1 year before CDK	4 (8.7%)	6 (11.11%)	0.75
CDK4/6i Ribociclib (*n* (%)) Palbociclib (*n* (%)) Abemaciclib (*n* (%))	24 (52.17%) 17 (36.96%) 5 (10.87%)	41 (75.93%) 10 (18.52%) 3 (5.56%)	0.05
Endocrine therapy Letrozole (*n* (%)) Fulvestrant (*n* (%))	29 (63.04%) 17 (39.36%)	36 (66.67%) 18 (33.33%)	0.84

Abbreviations: CHT—Chemotherapy; ECOG—Performance status assessed by Eastern Cooperative Oncology Group; mets—Metastases; CDK4/6i—Cyclin-dependent kinase 4 and 6 inhibitors; RT—Radiotherapy.

**Table 2:** Radiation therapy dose and fractionation.

Radiation Therapy	Concurrent (*n*)	Sequential (*n*)	Total (*n*)
**Palliative Setting**	**45**	**57**	**102**
8 Gy/1 fraction	17	18	35
20 Gy/5 fractions	19	30	49
30 Gy/10 fractions	7	8	15
Other (21 Gy/7 fractions 30 Gy/15fr, 10 Gy/5 fractions)	2	1	3
**Stereotactic radiation**	**16**	**16**	**32**
25 Gy/5 fractions	1	0	1
36–54 Gy/3 fractions	5	4	9
24–30 Gy/3 fractions	4	6	10
15–18 Gy/3 fractions	1	2	3
12 Gy/2 fractions	0	2	2
15–20 Gy/1 fractions	5	2	7
**Radical locoregional therapy**	**2**	**3**	**5**
50 Gy/25 fractions	0	2	2
42,5 Gy/17 fractions	0	1	1
45 Gy/20 fractions	2	0	2
**TOTAL**	**63**	**76**	**139**

**Table 3:** Radiation therapy sites.

Radiation Therapy	Concurrent (*n*)	Sequential (*n*)	Total (*n*)
Bone	49	59	108
Vertebrae	25	41	66
Cervival	5	7	12
Thoracic	8	15	23
Lumbar	10	10	20
>1 segment of the spine	2	9	11
Pelvis	11	11	22
Other bones (skull, sternum, extremities)	10	7	17
Multiple bone metastases	3	0	3
Central Nervous System	2	9	11
Breast/chest wall/regional lymph nodes	7	4	11
Lung/liver	2	2	4
Other soft tissues	3	2	5
**TOTAL**	**63**	**76**	**139**

## Data Availability

Core set with data of patients treated with radiotherapy and iCDK4/6 is available.

## Supplementary Materials

The following supporting information can be downloaded at: https://www.mdpi.com/article/10.3390/cancers15030690/s1, Table S1: Univariate regression analysis - Cox regression model for CDK4/6i dose reduction; Table S2: Multivariate regression analysis - Cox regression model for CDK4/6i dose reduction. Variables with p ≤ 0.2 in the univariate regression analysis were chosen for this analysis.
