# Supplementary material for: Safety and Feasibility of Radiation Therapy Combined with CDK 4/6 Inhibitors in the Management of Advanced Breast Cancer
Source: Cancers (Basel). 2023 Jan 22;15(3):690. doi: 10.3390/cancers15030690 (PMC9913652; doi:10.3390/cancers15030690)
Supplement: Supplementary file 1 [file cancers-15-00690-s001.zip › cancers-2133267-supplementary.pdf]

**Table S1.** Univariate regression analysis - Cox regression model for CDK4/6i dose reduction.

| Variable | | HR | 95 CI | P value |
|---|---|---|---|---|
| Age | <50 (ref) vs. ≥50 | 0.63 | 0.33-1.22 | 0.17 |
| ECOG | 0 (ref) vs. 1 | 2.22 | 1.07-4.61 | **0.03** |
| | 0 (ref) vs. 2 | 1.08 | 0.37-3.10 | 0.89 |
| Disease | De novo (ref) vs. Recurrent | 1.13 | 0.58-2.20 | 0.71 |
| Previous CHT | Yes (ref) vs. No | 1.21 | 0.64-2.29 | 0.56 |
| Line of treatment | First (ref) vs. Second | 0.93 | 0.46-1.89 | 0.85 |
| CDK4/6i | Rib (ref) vs. Palb | 1.06 | 0.53-2.11 | 0.88 |
| | Rib (ref) vs. Abem | 0.39 | 0.05-2.88 | 0.36 |
| RT | Concurrent (ref) vs. Sequential | 0.60 | 0.32-1.15 | 0.13 |
| RT area | Bone (ref) vs. Not-bone | 1.63 | 0.84-3.17 | 0.15 |
| Neutr in C1D14 | G0 (ref) vs. G1-2 | 1.85 | 0.87-3.97 | 0.11 |
| | G0 (ref) vs. G3-4 | 3.84 | 1.62-9.13 | **0.002** |
| Neutr in C2D1 | G0 (ref) vs. G1-2 | 2.29 | 0.65-8.07 | 0.20 |
| | G0 (ref) vs. G3-4 | 16.11 | 4.73-54.92 | **< 0.001** |

*Abbreviations: ref – reference; ECOG - Performance status assessed by Eastern Cooperative Oncology; CHT – chemotherapy; CDK4/6i - Cyclin-dependent kinase 4 and 6 inhibitors; RT – radiotherapy; Neutr – neutropenia: 0-no neutropenia; 1-neutropenia G1; 2-neutropenia G2; 3-neutropenia G3; 4-neutropenia G4; Rib – ribociclib; Palb – palbociclib; Abem – abemaciclib;*

**Table S2.** Multivariate regression analysis - Cox regression model for CDK4/6i dose reduction. Variables with p ≤ 0.2 in the univariate regression analysis were chosen for this analysis.

| Variable | | HR | 95 CI | P value |
|---|---|---|---|---|
| Age | <50 (ref) vs. ≥50 | 0.96 | 0.48-1.22 | 0.92 |
| ECOG | 0 (ref) vs. 1 | 3.13 | 1.37-7.12 | **0.007** |
| | 0 (ref) vs. 2 | 1.88 | 0.56-6.30 | 0.31 |
| RT | Concurrent (ref) vs. Sequential | 0.96 | 0.48-1.94 | 0.91 |
| RT area | Bone (ref) vs. Not-bone | 1.95 | 0.97-3.95 | 0.06 |
| Neutr in C1D14 | G0 (ref) vs. G1-2 | 0.38 | 0.14-1.00 | 0.05 |
| | G0 (ref) vs. G3-4 | 1.14 | 0.40-3.29 | 0.81 |
| Neutr in C2D1 | G0 (ref) vs. G1-2 | 2.99 | 0.82-10.94 | 0.10 |
| | G0 (ref) vs. G3-4 | 39.90 | 9.33-170.66 | **< 0.001** |

*Abbreviations: ref – reference; ECOG - Performance status assessed by Eastern Cooperative Oncology; RT – radiotherapy; Neutr – neutropenia: 0-no neutropenia; 1-neutropenia G1; 2-neutropenia G2; 3-neutropenia G3; 4-neutropenia G4;*
